# Correction to: Pyrethrin Biosynthesis: The Cytochrome P450 Oxidoreductase CYP82Q3 Converts Jasmolone To Pyrethrolone

**DOI:** 10.1093/plphys/kiad666

**Published:** 2024-01-10

This is a correction to: Wei Li, Daniel B. Lybrand, Fei Zhou, Robert L. Last, Eran Pichersky, Pyrethrin Biosynthesis: The Cytochrome P450 Oxidoreductase CYP82Q3 Converts Jasmolone To Pyrethrolone, Plant Physiology, Volume 181, Issue 3, November 2019, Pages 934–944, https://doi.org/10.1104/pp.19.00499

In the originally published version of this manuscript, in the Accession Numbers section, accession number MG874675 is incorrect and should have been given as MG874680. This section should, therefore, read as follows:

‘Accession Numbers

The sequence of TcPYS has been submitted to the National Center for Biotechnology Information under accession no. MG874680.'

These details have been corrected only in this correction notice to preserve the published version of record.

